# Adipogenic progenitors in different organs: Pathophysiological implications

**DOI:** 10.1007/s11154-021-09686-6

**Published:** 2021-10-29

**Authors:** Francesca Favaretto, Silvia Bettini, Luca Busetto, Gabriella Milan, Roberto Vettor

**Affiliations:** Department of Medicine, Internal Medicine 3, University of Padua, via Giustiniani 2, 35128 Padua, Italy

**Keywords:** Adipose tissue, Obesity, Adiposopathy, Adipose tissue-derived stromal and stem cells (ASCs), Mesenchymal stromal cells (MSCs), Intermuscular adipose tissue (IMAT), Signaling

## Abstract

In physiological conditions, the adipose organ resides in well-defined areas, where it acts providing an energy supply and as an endocrine organ involved in the control of whole-body energy metabolism. Adipose tissue adipokines connect the body’s nutritional status to the regulation of energy balance. When it surrounds organs, it provides also for mechanical protection. Adipose tissue has a complex and heterogenous cellular composition that includes adipocytes, adipose tissue-derived stromal and stem cells (ASCs) which are mesenchymal stromal cells, and endothelial and immune cells, which signal to each other and to other tissues to maintain homeostasis. In obesity and in other nutrition related diseases, as well as in age-related diseases, biological and functional changes of adipose tissue give rise to several complications. Obesity triggers alterations of ASCs, impairing adipose tissue remodeling and adipose tissue function, which induces low-grade systemic inflammation, progressive insulin resistance and other metabolic disorders. Adipose tissue grows by hyperplasia recruiting new ASCs and by hypertrophy, up to its expandability limit. To overcome this limitation and to store the excess of nutrients, adipose tissue develops ectopically, involving organs such as muscle, bone marrow and the heart. The origin of ectopic adipose organ is not clearly elucidated, and a possible explanation lies in the stimulation of the adipogenic differentiation of mesenchymal precursor cells which normally differentiate toward a lineage specific for the organ in which they reside. The chronic exposition of these newly-formed adipose depots to the pathological environment, will confer to them all the phenotypic characteristics of a dysfunctional adipose tissue, perpetuating the organ alterations. Visceral fat, but also ectopic fat, either in the liver, muscle or heart, can increase the risk of developing insulin resistance, type 2 diabetes, and cardiovascular diseases. Being able to prevent and to target dysfunctional adipose tissue will avoid the progression towards the complications of obesity and other nutrition-related diseases. The aim of this review is to summarize some of the knowledge regarding the presence of adipose tissue in particular tissues (where it is not usually present), describing the composition of its adipogenic precursors, and the interactions responsible for the development of organ pathologies.

## The pathological expansion of adipose tissue throughout the body

Obesity is recognized as a multifactorial chronic disease in which the qualitative and quantitative changes of the histological architecture of the adipose organ and the consequent functional alterations account for the severity of the disease and the development of its complications [1–3]. The primary cause of obesity is likely an altered relationship between the environment and the genetic heritage of the control systems which is at the basis of the regulation of energy metabolism and influences the bidirectional pathways of the energy transfer between the environment and the body [4]. Diverse evolutionary changes acting on the nuclear, mitochondrial and microbiota genome may impact genetic predisposition to obesity [5]. Obesity can also be considered an intrinsic path represented by the alteration of the dialogue between the center and the periphery or between the control systems present at the level of the Central nervous system (CNS) and the energy depots of the body, where the adipose organ is the most important source [6, 7].

Obesity and adipose organ dysfunction are the trigger of several complications such as cardiovascular disease, type 2 diabetes (T2D), and cancer, whose pathophysiological bases are very complex, in which the metabolic, hormonal, inflammatory and immune aspects coexist along with alteration of cellular composition and between cell communication [8, 9]. Adipose tissue (AT) is composed not only of adipocytes and their precursors, but also of an array of immune cells and an exclusive extracellular matrix, whose behavior under acute and chronic hypercaloric states is quite different [10].

**Fig. 1:**
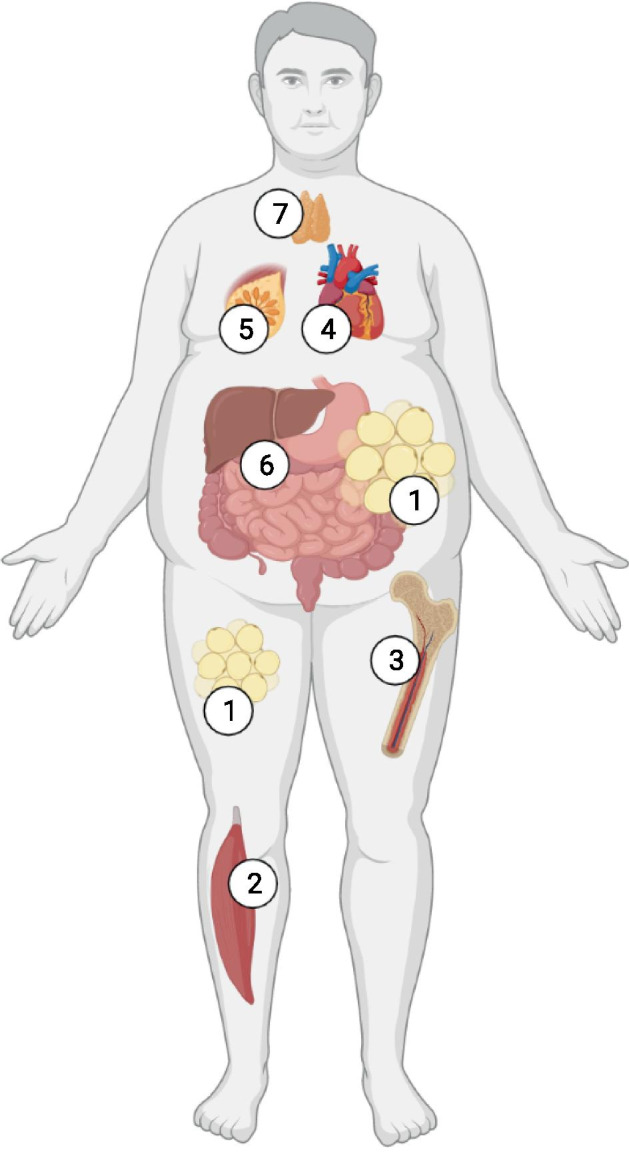
Pathological adipose tissue deposition. In humans during obesity and ageing, adipose tissue (AT) increases in mass, both as subcutaneous (SAT) and visceral (VAT). Moreover, it accumulates also outside from its physiological conserved regional location. The expansion nearby others organs cause their functional impairment and the appearance of comorbidities. (1) subcutaneous and visceral AT; (2) intermuscular AT; (3) bone marrow AT; (4) pericardial AT; (5) mammary AT; (6) mesenteric AT; (7) thymic AT. Created with BioRender.com

The adipose organ could be defined as a diffuse organ and in fact not only does it exist in anatomical structures that are easily dissectable and separable from the context of other organs [11], but it can also be located both within organs in which physiologically it should not be [12], or surround organs and tissues in anatomical continuity or be clearly distinguished from them by anatomical borders (Fig. 1). As discussed by Zwick et al. [13], AT could assume different, often non-traditional functions depending on its proximity to other organs in relation to their physiological and pathological conditions. Moreover, in stem cell-rich skin, bone marrow, and mammary glands, adipocytes signal to and modulate organ regeneration and remodeling. In mammary glands and heart, adipocytes supply lipids to neighboring cells for nutritional and metabolic functions, respectively. AT near the surface of skin and intestine senses and responds to bacterial invasion, contributing to the body’s innate immune barrier. As the recognition of diverse adipose depot functions increases, novel therapeutic approaches centered on tissue-specific adipocytes are likely to emerge for a range of cancers and regenerative, infectious, and autoimmune disorders.

The purpose of this review is to consider the pathogenetic interactions between the presence of AT in particular sites, and in any case where it is usually not present, and the development of organ pathology such as sarcopenia and osteoporosis.

## Adipose organ and the pathophysiological basis of adiposopathy

AT grows by hypertrophy, which consists in the enlargement of cell size by accumulation of triglycerides (TG) in lipid droplets, and by hyperplasia, recruiting and differentiating adipose progenitors into new mature cells [14]. While the first process refers to a metabolic adaptation of a single adipocyte to the environmental excess of nutrients, lipids in particular, and allows rapid and reversible change in AT mass, the second involves many cell types and their coordinated interactions and causes an increase in the number of adipocytes changing the functional properties of the resulting new AT. Both biological processes have an impact on the surrounding extracellular matrix, that influences them, in turn, through its composition and structure and thanks to the presence of other cell types.

In particular AT is crossed by many vessels supplying nutrients, oxygen and signal molecules to mature adipocytes [15], contains several nervous fibers cooperating to the functional shaping and tuning of the tissue [16], and includes some immune cells [17] and progenitor cells near to the capillary structures [18]. All these elements actively contribute to the healthy AT growth and functioning that are different and specific depending on the tissue color (white, brown and beige AT) and its anatomical localization (visceral, VAT, vs subcutaneous, SAT). In this way AT is able to efficiently fit the ever-changing environmental conditions (such as nutrient availability and temperature levels).

Adipose tissue stromal and stem cells (ASCs) have been isolated and characterized from both mice and humans proving their capacity to give rise to different lineages of mesenchymal origin *in* *vitro* and *in* *vivo* [19, 20]. In particular several researchers contribute to identify their surface markers and tissue localization by means of gold standard techniques such as cytometry, IHC and IF [21]. ASCs belong to the Mesenchymal stromal cells (MSCs) population and have been compared with Bone Marrow and Umbilical –derived stem cells especially for their immunomodulatory properties in the clinical trial setting [22, 23]. In humans, the combination of a few robust markers (CD34 + /CD31−/CD45−) allows for identifying and quantifying the ASC population ex-vivo to study its role in different pathological conditions [24]. Several studies, mainly conducted on animals, evidence a possible hierarchical relationship between ASCs, and suggest the existence of white, brown and beige precursors as well as depot-specific subpopulations of ASCs [25, 26]. Recent studies and new single-cell analysis underline the heterogeneity of ASCs describing new regulatory subpopulations controlling the AT expansion (ASC reg) [18]. The increasing data make the picture more complicated, but greatly enhance the range of possible therapeutic interventions.

The term “adiposopathy” indicates the disease of AT starting from damage to one or more of its components and causing a dysfunction of its metabolic and endocrine function [27], growth capacity and plasticity [28, 29]. Certainly, adiposopathy plays an important role, and according to some authors represents the primum movens in the genesis and development of obesity, a complex disease associated with several metabolic alterations involving different organs, tissues and cell types.

Two of the major signs of adiposopathy are represented by fibrosis and inflammation, which are well documented and deeply characterized in AT. Fibrosis is associated with a dramatic change in the composition and the physical characteristics of the AT extracellular matrix that affects several morphological and functional AT properties and is strongly linked with inflammation. Fibrosis increases in obesity and metabolic complications, both in animal models and humans, and probably involves the activation and proliferation of specific precursor cells (PDGRFα + /CD9hi) [30].

Many immune cells in AT have been described as participating in obesity-associated inflammation, thus obesity should be regarded as an inflammatory disease. Among them macrophages constitute a large proportion, are characterized by a pro-inflammatory M1-like phenotype and are recruited and activated in the AT by inflammatory cytokines and chemokines which are also secreted by dysfunctional adipocytes. Recently the phenotype plasticity of these macrophages has been thoroughly investigated, underlining the link between fuel bioenergetics and polarization, and evidencing their role in modulating lipid, catecholamine and iron availability [31]. Several T cell populations CD4 + (Th1, Th17, Tregs), CD8 + and iNKT are identified as resident immune cells in AT, especially in animal models, with specific pro-inflammatory or anti-inflammatory roles in obesity [17].

We recently showed that people with obesity, characterized by higher Body mass index (BMI), waist and LDL blood level than controls, displayed adipocytes larger in size and a higher number of ASCs both in VAT and in SAT compared to normal weight subjects, attesting that their white AT, independently of its anatomical localization, is able to expand by hypertrophy and hyperplasia to accommodate the excess nutrient intake. The number of capillaries per mm^2^ present in their AT is reduced compared to controls, probably due to the enlargement of the adipocytes rather than to a reduction in the angiogenic potential. Similar histomorphological evaluations performed on people with obesity and with a concomitant presence of early signs of metabolic complication (prediabetic condition) or stable diagnosed T2D, clearly showed that AT continues to expand by hypertrophy but does not sustain the new adipogenesis decreasing the number of ASCs [32]. Other studies performed both in animal models and human samples showed that hypertrophic adipocytes displayed alterations in metabolic and endocrine functions, expressing and secreting a different adipocytokine pattern with pro-inflammatory and pro-fibrotic effects [33]. Evidence has been collected so far indicating that while AT can preserve its adipogenic potential giving rise to new metabolic healthy small adipocytes, its expansion can be positive and functional [14]. In fact, a decrease in the AT stemness always corresponds to a hazardous unbalance in the growth, giving rise to lipid spillover from AT, alterations of AT endocrine signals and ultimately the development of metabolic complications.

## Skeletal muscle and the origin of intermuscular adipose tissue

Skeletal muscle changes are not uniformly observed in individuals with obesity and the presence of heterogeneous phenotypes may contribute to their underestimation [34]. Indeed, a positive association between BMI and lean body mass has been reported in general population studies, and moderate increments in skeletal muscle mass may occur in obesity as a consequence of higher postural and ambulatory muscle work as well as potential direct anabolic effects of higher dietary protein intake. However, it is increasingly clear that profound skeletal muscle metabolism changes may occur in some people with obesity and may lead to altered body composition with higher fat mass and substantial impairment of muscle mass and quality [34].

Low muscle function and mass are currently addressed in obese individuals under the definition of sarcopenic obesity. The latter is based on the originally geriatric concept of sarcopenia, i.e. the age-associated combination of declining muscle mass and function (particularly muscle strength) [35]. The concept of sarcopenic obesity has been primarily applied in elderly people with obesity, but increasing evidence suggests that muscle impairment could occur at any age in individuals with obesity in the presence of metabolic complications, chronic comorbidities, acute or critical illness (including cancer), and following bariatric surgery or strict hypocaloric diet [34].

Despite the fact that common shared diagnostic criteria for sarcopenic obesity are still lacking [36], several alterations in skeletal muscle have been described [34]. In particular, lipid accumulation in skeletal muscle cells is considered a typical feature and it has been closely linked with tissue and systemic insulin resistance [37]. Mechanisms mediating metabolic lipotoxicity in skeletal muscle are complex, including direct pro-oxidative and inflammatory activities [38] as well as accumulation of metabolically toxic lipid moieties such as diacylglycerol and ceramides [39]. Lastly, ectopic lipid deposition may also compromise muscle protein turnover [40], promoting an ‘anabolic resistance’ state in which the response of skeletal muscle protein synthesis to nutrients is blunted and loss of muscle mass is promoted [34].

Parallel to lipid accumulation into myocells, the skeletal muscle of people with sarcopenic obesity is characterized by the “infiltration” of the muscular tissue by an increasing amount of adipocytes. The origin of the “new” adipocytes appearing in the muscle is still debated and several hypotheses have been made. Among the different mechanisms that could be responsible for the accumulation of fat in this site, the dysdifferentiation of muscle-derived stem cells or other mesenchymal progenitors has been postulated, turning them into cells with an adipocyte phenotype. In particular, muscle satellite cells (SCs) can acquire features of adipocytes, including the abilities to express adipocyte-specific genes and accumulate lipids. Failure to express the transcription factors that direct mesenchymal precursors into fully differentiated functionally specialized cells may be responsible for their phenotypic switch into the adipogenic lineage. Human SCs also possess this adipogenic potential, thus explaining the presence of mature adipocytes within skeletal muscle. Several pathways and factors (PPARs, WNT growth factors, myokines, GEF-GAP-Rho, p66(shc), mitochondrial ROS production, PKCβ) could be implicated in the adipogenic conversion of SCs [41–46]. Moreover, age, oxidative stress, injury, and regeneration can also activate adipogenic differentiation potential in myoblasts affecting fat accumulation in skeletal muscle [47]. This activation of the transdifferentiation of myoblasts into adipocytes can be mediated by several transcriptional factors (like Peroxisome proliferator‐activated receptors (PPARs), PR domain‐containing protein 16 (Prdm16), deletion of MyoD, Bone morphogenetic proteins (BMPs) and others), by the expression of specific MicroRNAs (miRNAs), and by nutritional factors [47]. The switch of muscular precursors from the myogenic to the adipogenic lineages of differentiation can be modulated also by the complex interplay between several interstitial cell types in the regenerating skeletal muscle. For instance, an interstitial CD142-positive cell population has recently been identified by single-cell RNA sequencing in mice and humans responsible for the inhibition of adipogenesis through GDF10 secretion [48]. This CD142-positive cell type is nearly absent in muscular dystrophy, where the interstitial cell composition is completely altered, muscle-cell regeneration is impaired and adipocytes abundant [48]. Moreover, adipogenesis was observed in Fibro-adipogenic cells (FAPs) [49, 50] which are muscle-resident non-myogenic progenitors of mesenchymal origin that proliferate in response to injury and are involved in muscle loss [51].

Alternatively to aberrant adipogenic differentiation of muscle stem cells, the adipocytes infiltrating the skeletal muscle in sarcopenic obesity could be derived by migration of adipocyte precursors from AT. Subcutaneous adipose tissue is able to release ASCs into circulation, under the regulation of the chemokine CXCL12 and its receptor CXCR4 [52]. Triggering the release of adipocyte precursors from subcutaneous AT with a high-fat diet and exposure to a CXCR4 antagonist directly promoted ectopic adipocyte formation in skeletal muscle in mice [53]. These data provide insights into the role of adipocyte progenitor trafficking in promoting ectopic fat deposition.

In conclusion, lipid accumulation and adipocyte infiltration in skeletal muscle is a part of the picture of sarcopenic obesity, typically occurring with aging or in the presence of metabolic diseases. These alterations in muscle structure have an important role in explaining insulin-resistance and loss of muscle strength and performance.

## Adipose tissue in other organs

### Bone marrow

AT localizes in Bone marrow (BM), where it is called Marrow adipose tissue (MAT) and exists in two forms: constitutive (cMAT) and regulated (rMAT), depending on the ability to respond to external stimuli. The first one is mainly present in the distal skeleton (yellow marrow) and its adipocytes develop soon after birth, possess high levels of CEBPA (CCAAT enhancer binding protein alpha) and CEBPB (CCAAT enhancer binding protein beta) and are filled by mono-unsaturated fatty acids. rMAT -adipocytes (which are relatively small) are enriched in the proximal, hematopoietic skeleton within the red marrow and form more gradually throughout life and expand or reduce in response to endogenous and exogenous stimuli, accumulating saturated fatty acids. The mass of MAT increases in response to pathological conditions, including aging, obesity, T2D and osteoporosis and during radiotherapy or glucocorticoid treatment [54].

Histological assessments and imaging methods describe that MAT in adult humans ranges from 30 to 70% of BM volume and collectively can also reach 8% of total body fat mass [54]. Although the unilocular adipocytes populating the BM were thought to be acting as passive space fillers for a long time, nowadays it is clear that they possess their own molecular signature, do not share any precursors with WAT and BAT and are functionally different from them [55].

Its high representation explains the multiple functions played in the regulation of systemic homeostasis of energy and endocrine mediated by adipokines such as leptin and adiponectin production [56, 57], by the release of free fatty acids [58] or by the regulation of basal glucose uptake [55].

The BM-MSCs are self-renewing and give rise to osteoblasts, chondrocytes and adipocytes [59]. Obviously, an extensive characterization of MAT adipocytes has been carried out, but a precise lineage hierarchy is still lacking. Lineage tracing analyses were not able to clarify whether MAT adipocytes arise from a single population or from a small number of different progenitors in the BM [60–62].

The tight constraint between cells in the BM microenvironment explains the influence of MAT on hematopoiesis and skeletal remodeling [63].

MAT is involved in the regulation of renewal, quiescence and differentiation of Hematopoietic stem cells (HSC) which provide for myeloid and lymphoid progenitors [64]. Its increase during ageing and a high-fat diet seems likely to be related to an increased myelopoiesis in association with a reduced lymphopoiesis [65, 66]. *In* *vitro* experiments demonstrated that the impairment linked to B-lineage progenitors is exerted by multiple actors: adipocyte-derived soluble factors, IL-1 and activation of inflammosome [67–69]. The switch toward myeloid populations induces tissue infiltration of monocytes and their differentiation into proinflammatory macrophages that prompt tissue inflammation and worsen metabolic disease [70]. In obesity, MAT is positively associated to ectopic fat deposition, serum lipid levels [71], and VAT [72–74]; it also increases with ageing in people with osteoporosis [75], and pelvic MAT has been inversely correlated with bone volume in younger and older adults [76, 77]. However, MAT displays a dynamic regulation involving the mineral metabolism [78]. Two possible theories have been supported by experimental findings: one is that a common BM progenitor (Osterix + /endosteal and/or LepR + /sinusoidal) is pushed toward adipogenesis at the expense of osteogenesis, sustaining a local feedback loop exacerbating abnormalities [61, 62]. Another possible explanation is that MAT’s endocrine properties change during pathological conditions modifying secreted factors (e.g., RANKL, CXCL1, CXCL2, IL-6, G-CSF) that directly regulate bone remodeling through fine tuning of the osteoblast-osteoclast activity [57], even in the presence of minor alterations in MAT mass.

## Others

### Heart

The mediastinal or intrathoracic VAT is classified as epicardial (EAT), paracardial (PAT), pericardial (which is epicardial plus paracardial), perivascular (PVAT) and intramyocardial AT. In normal physiological conditions, the EAT provides Free fatty acids (FFAs) to myocardium demands, it secretes vasoactive factors (i.e., leptin, adiponectin, nitric oxide, omentin) and it has thermogenic properties, expressing markers typical of the BAT (i.e., uncoupling protein 1, UCP-1). Several clinical studies have demonstrated a close association between the EAT and the occurrence of Coronary artery disease (CAD) [79] and Heart failure with preserved ejection fraction (HFpEF) [80]. The EAT volume, quantified by computed tomography, correlates with both the presence and the extent of calcified coronary artery plaques [81, 82], and is an independent predictor of early CAD [83]. EAT thickness is significantly correlated with obesity, insulin resistance, waist circumference, blood pressure, sleep apnea severity and dyslipidemia, suggesting that this AT depot could be considered an indicator of cardiovascular risk [84]. Indeed, caloric restriction and bariatric surgery has been demonstrated to reduce EAT [85], thus weight loss in obesity could be targeted to reduce EAT in order to obtain cardiovascular prevention. Coronary arteries are surrounded by PVAT, representing a mechanical support and being a vasocrine and paracrine source of cytokines and adipokines, it also participates in vascular remodeling. Recently, a new imaging approach has been developed for evaluating PVAT inflammation as a determinant of acute plaque rupture [86]. One of the possible involved mechanisms linking EAT and CAD is proximity, whereas the atherosclerotic lesions were preferentially localized in coronary arteries surrounded by AT. This closeness allows for a paracrine EAT-myocardium crosstalk, by direct diffusion of proinflammatory cytokines and other adipokines [87]. Conversely, cytokines from the EAT may be directly released into the vasa vasorum of the coronary arterial wall [88]. Consequently, it was hypothesized that EAT dysfunction, with a great number of inflammatory cells and a high expression of inflammatory cytokines, induces inflammation of adjacent arteries and thus contributes to the pathogenesis of atherosclerosis, plaque rupture, and thrombosis [89]. Several reports tried to address to the Cardiac adipocytes (CAs) origin-question, and recently it was demonstrated that PDGFRa^+^ and PDGFRb^+^ cardiac Mesenchymal cells (MCs) give rise to the intramyocardial CAs during development [90]. As in muscle, mesenchymal progenitors give rise to FAPs also in the heart [91] and cardiac stromal fibroblasts have been recognized as covering an important role both in health and disease. Acute ischemic injury or age seem to promote only fibrogenic differentiation, conversely, obesity pushes both fibrotic and adipogenic programs in FAPs [92].

### Thymus

The thymus is one of the pillars of adaptive immunity. At the time of puberty, after an active period of production of new T-cells, the thymus loses the stroma and fills with fat (thymic AT, TAT). This involution decreases T-cell response to antigens. Conversely, the antigen-independent proliferation of T-cell is maintained and it may lead to dysfunctional cells, secreting proinflammatory factors [93]. Obesity accelerates the age-related reduction of T-cell receptors (TCRs). In fact, diet-induced obesity reduces thymocyte counts and significantly increases apoptosis of developing T-cell populations. Moreover, the TCR spectra typing analysis showed that obesity also compromised TCR repertoire diversity [94]. Indeed, it was demonstrated that leptin runs a thymoprotective role, not directly but rather as a secondary effect of suppression of AT expansion [95]. Lastly, the ASCs have been shown to increase expression of immunosuppressive markers in the thymus, preventing lymphocyte differentiation and promoting survival of T cells in a quiescent state [96].

## Peritumoral adipose tissue

ASCs have a crucial role in cancer [97]. They can be chemoattracted to solid cancers and secrete several cytokines in a paracrine fashion, among them IL6 and Vascular endothelial growth factor (VEGF), which are able to promote the inflammatory microenvironment and further increase tumorigenesis, nutrient and oxygen metabolism [98], thus increasing cancer proliferation, survival and spreading.

Emerging evidence indicates a link between obesity and cancer progression [99]. Tumor microenvironment (TME) is defined as a unique metabolic niche, containing tumor, immune and stromal cells. Age-related immune deterioration is exacerbated by obesity in animal and human studies and impacts also on the metabolic landscape of TME. It was suggested that peritumoral AT (cancer associated adipocytes, CAAs) contributes to tumor initiation, growth and invasion in different kinds of tumors [100, 101].

Furthermore, peritumoral AT may promote, by paracrine signaling, the expression of depot-specific factors associated with therapeutic resistance [102]. A significant decrease in T cell proliferative capacity and a significant deficit in IFNγ and Tumor necrosis factor (TNF) production in diet-induced obese mice has been reported. This mechanism is partially mediated by leptin [103]. It was shown that a high-fat diet accelerates tumor growth and reduces the number and the function of intratumoral CD8 + T cells in mice due to altered fatty acid portioning and local depletion of essential metabolites [104].

## Autocrine, paracrine and endocrine crosstalk alterations

The biological complexity of obesity is centered on information-based intricacy: the disruption of the information system controlling food intake and energy expenditure as well as the inter-organ crosstalk or dialogue could be seen as the major determinant in the obesity pathogenesis [2, 3, 6]. The fundamental nature of the communication process is characterized by the storage, transmission, and utilization of information, coding and decoding of the signals and their nature or origin [105, 106]. One of the principal contributions in the last three decades to the field of communication has been in the area of cybernetics, from which information theory is derived [107]. It can be considered today, together with artificial intelligence, as a system that acts to achieve a goal [108]. Cybernetics is the science of the feedback system, such as maintaining the temperature level or blood glucose concentration [109]. The system takes action to correct the differences between the current state and the goal through feedback signals. This process helps ensure stability when the perturbations threaten second order dynamic systems, which, observing the results of this perturbation within themselves, can change their goals. Second-order systems don’t just react; they can also learn, activating amongst them a conversation or a dialogue, an exchange on objectives and means.

It is possible to recognize in the various organs that make up the human body the single sections of an orchestra. The orchestra needs a code represented by the score to which each individual instrument refers, and also a conductor who moves all the sounds of the various instruments in a coordinated manner. The communication between the conductor and the sections (inter-organ crosstalk) and of the musicians amongst themselves (intra-organ crosstalk) is fundamental to harmonize every single note in a symphony. It is important to try to decode these communication systems to understand how homeostasis is preserved.

One of the most relevant reasons for a preferential inter-organ crosstalk is based on the developmental common origin of AT muscle and bone. Mesenchymal stem cells give rise to three main cell lineages by an early differentiation in osteoblast, adipoblasts and myoblasts which represent already committed stem cells of the three specific lineages which lead to the mature cell phenotype of osteocyte, adipocyte and myocyte [110].

**Fig. 2:**
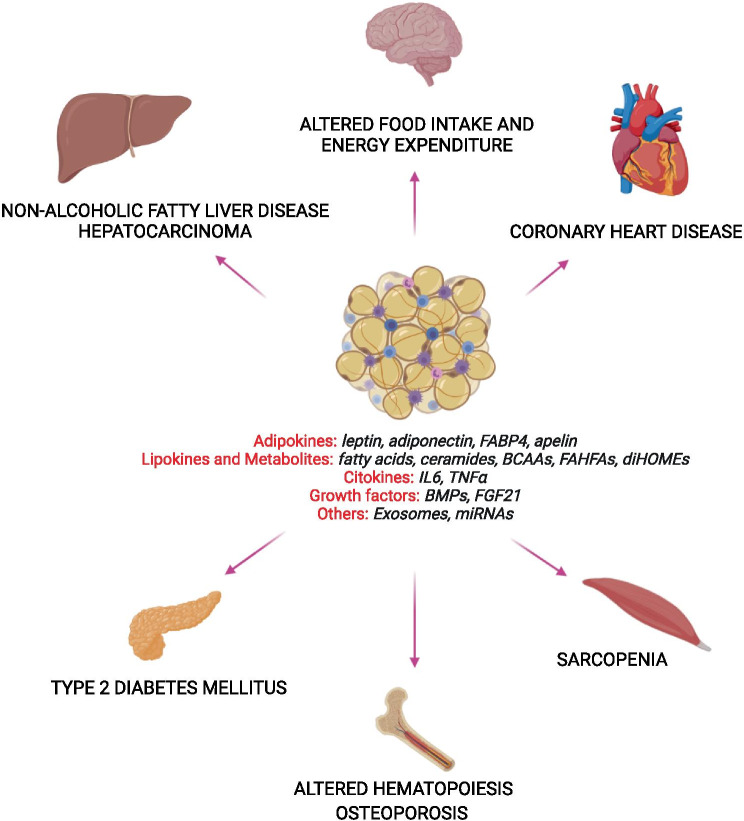
Pathological adipose tissue endocrine signals. Adipose tissue synthesizes and delivery a complex array of molecules including cyto/adipokines, metabolites and lypokines, growth factors, extracellular vesicles (exosomes) and miRNAs which flow throughout the bloodstream and reach the target organs. The pathological increase of adipose tissue induces qualitative and quantitative changes in the production of its signals, provoking an overall alteration of the long-range communication, ending in the impairment of multiple organs and their functions. *FABP4* fatty acid-binding protein 4, *BCAAs* branched-chain aminoacids, *FAHFAs* fatty acid esters of hydroxy fatty acids, *diHOMEs* dihydroxy octadecenoic acids, *IL6* interleukin 6, *TNFα* tumor necrosis factor alpha, *BMPs* bone morphogenetic proteins, *FGF21* fibroblast growth factor 21. Created with BioRender.com

AT, besides its ability to store blood nutrients as glucose and fatty acids, releases plenty of peptides, collectively called adipokines, and several metabolites as lactate and fatty acids and lipid signals / mediators (Fig. 2). The flux of lipid substrates to the adipose cell is a sensing mechanism of an increased availability of energy, transduced by specific receptors or transporters and evoking the release of leptin which transfers this information to the target hypothalamic structures [111–113]. The preferential channeling of fuel substrates toward AT rather than muscle could be determined by the decrease in oxidative capacity of skeletal muscle because of a sedentary lifestyle, aging or mitochondrial or genetic alterations [114]. This overflow of substrate continues until the expandability capacity of the adipose organ has been overcome due to the appearance of fat cell insulin resistance, when both long-term and short-term regulation and delivery of stored lipids in response to the peripheral energy need is no longer finely controlled and qualitative and/or quantitative changes in adipokine production are triggered.

**Fig. 3:**
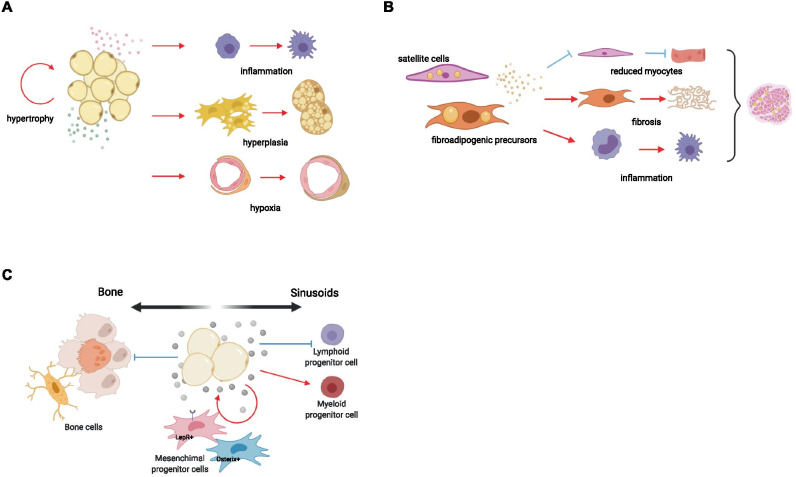
Pathological paracrine and autocrine signals mediated by adipocytes worsen functional and structural tissue remodeling. **A** Secreted adipocyte-factors (different color spheres) act on the diverse cellular component of adipose tissue. Pathological adipocytes induce recruitment and activation of macrophages causing a pro-inflammatory microenvironment. When they engage adipose progenitors, promote the differentiation of new mature cells inducing hyperplasia. During diabetes, the reduced ability to store glucose by dysfunctional adipocytes, increase capillary basement membrane thickness favoring hypoxia. Finally, adipocytes can directly signal to themselves promoting hypertrophy. **B** During obesity or ageing adipose tissue appears in muscle, involving satellite cells (SCs) conversion to adipocytes and recruiting fibroadipogenic precursors. These are able to reduce the differentiation of SCs in myocytes, activate a fibrotic program and to enroll inflammatory cells, all these events in turn induces muscle loss and reduced strength. **C** Pathological signals released by bone marrow adipocytes act on hematopoietic stem cells, impairing their self-renewal and differentiation abilities, promoting myelopoiesis. In the meantime, adipocytes stimulate their accumulation recruiting mesenchymal stromal cells toward new adipogenesis at the expense of osteogenesis. In addition, osteoblast and osteoclast activities are directly altered by bone marrow adipocytes which cause bone remodeling. Red arrow: activation, Blue arrow: inhibition, Created with BioRender.com

In AT, autocrine and paracrine communication between the different adipocyte phenotypes, vasculo-stromal cells and immune cells could be of crucial importance in controlling AT biology, hypoxia, low‐grade chronic inflammation and fibrosis [115] (Fig. 3).

The accumulation of lipids and AT in skeletal muscles alters the secretion of several myokines acting both locally (IL-4, IL-6, VEGF, IGF-1 and irisin) and systemically (GDF8/myostatin, FGF21 and IL-6) which mediate the beneficial effects of exercise on other organs [116–118] and are involved in the regulation of energy homeostasis by AT itself, such as thermogenesis activation and lipid mobilization [119]. Moreover, a progressive decline in muscle myokines may contribute to the development of aging-related sarcopenia [120], as in the case of apelin which regulates muscle hypertrophy. In sarcopenic obesity the increased the presence of fat within skeletal muscle with structural and functional impairment of muscle may also affect bone density leading to the presence of osteoporosis. Myostatin, not only decreases muscle mass [121], but also negatively regulates bone mass. In adults, irisin was also (negatively) associated with vertebral fragility fractures [122] and sarcopenia in post-menopausal women, [123] suggesting that irisin plays a crucial role in bone metabolism to a greater extent when compared with its original effect on AT.

Osteocalcin (OCN) is a protein secreted in a carboxylated form (cOCN) by osteoblasts and is responsible for the matrix mineralization and bone formation, while the undercarboxylated form (ucOCN) controls numerous physiological processes in an endocrine fashion [124]. People who are overweight and people with obesity had a lower ucOCN and ucOC/OCN ratio negatively correlated to BMI. OCN is also expressed and released by subcutaneous and omental AT and during all stages of adipogenesis [125]. OCN is able to influence AT function by inducing adiponectin expression in adipocytes [125, 126]. The elevation in osteocalcin increases in humans during physical activity [127], increasing fatty acid transporter gene expression and β-oxidation, and promoting glucose uptake. During ageing its levels decrease, but experimental data displayed that the hormone prevents muscle loss regulating the protein turnover [128]. Bone and skeletal muscle could be considered as cooperating as a single functional unit. The presence of osteoporosis often parallels sarcopenia in many chronic diseases including obesity [34, 36], the decline in muscle function probably being the principal factor leading to the loss of bone mass. Moreover it cannot be excluded that the major trigger in inducing this dual impairment could be the development of adiposophaty and even more importantly the presence of ectopic AT within skeletal muscle and the excessive growth of AT inside the bone marrow, giving rise to a whole body or local autocrine/paracrine cytokine storm, supporting once again the hypothesis of inflammaging and metaflammation in the development of aging related diseases including osteoporosis and sarcopenia [129]. The mechanical loading due to the increased BMI results in increased bone mineral density, but its putative protective effect against fractures is not present in all skeletal sites, moreover it is well known that sarcopenic obese subjects display a reduction in bone mass. The relationship between obesity and bone health is complex and not completely elucidated. Obesity is thought to affect bone health through a variety of mechanisms such as mechanical loading, obesity phenotype, the distribution of AT, the secreted cytokines, gender, age, and bone sites. In fact, the risk for some non-vertebral fractures is higher, whereas the risk of vertebral and proximal femur fractures is lower in individuals with obesity as compared with subjects with normal weight [130, 131]. Obesity can initially increase bone mass, but later bone formation is unable to maintain the bone causing a reduction in its quality over the time [132].

## Concluding remarks

Adiposopathy or adipose organ dysfunction, whose hallmarks are represented by qualitative and quantitative changes in fat depots whether it is subcutaneous or visceral, white or brown, leads to and resides also in the ectopic AT. The main features are characterized by changes in the localization and morphology (anatomical distribution, the brown to white fat differentiation, increased of adipocyte size, fibrosis), function (decreased insulin sensitivity, failure of the lipid storage rising peripheral lipotoxicity, mitochondrial impairment), secretion (altered adipokine production) and cellular composition (reduced neoangiogenesis, macrophage infiltration, stem cell abnormalities), which appear stepwise [115].

It is possible to think that the continuous overflow of energy substrates, once the storage capacity and the expandability property of AT have been overcome, could ultimately result in a lipid infiltration within organs such as the liver, muscle, and heart or evoke the adipogenic differentiation of cell precursors or adult stem cells normally able to differentiate toward lineage-specific cell types of the tissue or organ in which they reside, regulating homeostasis, regeneration, and aging of all tissues. The chronic exposition of these newly-formed adipose depots (mainly WAT) to a pathological environment, will confer to them all the phenotypic characteristics of a tissue affected by adiposopathy, perpetuating the organ damage. Adult stem cell senescence has emerged as an attractive theory for the decline in mammalian tissue and organ dysfunction of a variety of cardiovascular and metabolic diseases [133–136]. In this view, a better characterization of human AT progenitors and the precise definition of their recruitment and regulation could improve the possibility of targeting specific pathways acting in the AT expansion [28, 137, 138]. A further fascinating mechanism explaining the origin and growth of ectopic AT could be the colonization by ASCs of other organs, such as muscle or bone marrow. It is likely that this could happen because of the common embryological origin of the three tissues. The majority of adipocytes originate from the mesoderm and are organized into anatomically distinct depots. ASCs play important roles in tissue regeneration, remodeling, and homeostasis and are able to differentiate into different lineages, such as adipocytes, cardiomyocytes, osteoblasts, chondrocytes and myocytes [139, 140]. Recently, it was demonstrated in mice that the release of ASCs from subcutaneous AT directly affects ectopic adipocyte deposition in muscle, and ongoing studies focus on demonstrating that this mechanism is not restricted to skeletal muscle, suggesting that AT could bypass its expandability limitation allowing the colonization of non-AT organs, to store lipids in a competent cell [53].

Pathological conditions such as aging, as well as congenital lipodystrophy, and, to some extent, even obesity in humans, are accompanied by a reduction or loss or limitation in the expandability of fat with an accumulation of adipose cells and lipids in non-adipose depots, such as bone marrow, liver, and skeletal muscle. This accumulation worsens the functional performance of the target organs, especially in muscle and bone. For both, mass and strength decline with aging and evidence suggests that many of the factors observed to stimulate bone marrow adipogenesis (leptin deficiency, disuse atrophy, lack of estrogen and glucocorticoid treatment) also induce myosteatosis [141]. In the same way, many of the conditions that induce marrow adipogenesis and bone loss in men and women also stimulate the accumulation of adipocytes and Intramyocellular lipid (IMCL) in skeletal muscle [142]. Ectopic AT accumulation represents a predictor of organ disfunction, for example IMAT defines muscle metabolism and function but it also seems a modifiable risk factor, therefore, the clarification of the mechanisms at the basis of ectopic fat formation may provide new potential targets in the treatment of metabolic diseases. At present, exercise and physical activity appear to be effective countermeasures against the accumulation of AT, even if it is not currently possible to completely revert the effects of aging [143, 144]. Moreover, calorie restriction may also reduce fatty infiltration and potentiate the effect of exercise [145]. Pharmacological intervention combined with lifestyle changes to delay or reverse myosteatosis is still largely unexplored, for instance, treatment with an antimyostatin antibody capable of blocking myostatin signaling to treat people with muscle loss, but its effect on IMAT loss is still far from being considered an effective therapeutic strategy [146].

In conclusion, adipose tissue within as well as surrounding crucial organs has been identified as an important determinant of target organ dysfunction. Its stemness and composition, secretome, and location define its function in health and metabolic disease (Table 1). Preventing the appearance of dysfunctional AT is certainly the prerequisite to avoiding the progression towards the most important complications of obesity and other nutrition related diseases.

**Table 1:** Summary of the main topics discussed in the review

**Regional distribution**	**Adipose tissue localization (acronym)**	**Pathological implications**	**Adipocyte progenitors**	**Progenitor markers**	**Signalling molecules**
**Adipose organ**	SAT, VAT, BAT	Adipose tissue dysfunction (Adiposopathy) and peripheral lipotoxicity, T2D, NAFLD/NASH, CVD	ASCs	CD34 + CD73 + CD90 + CD31–CD45-[32]	Leptin, adipocytokines, FFAs
**Muscle**	IMAT, IMCL	Altered metabolic, mobility function, and strength. Sarcopenia, IR	SCs FAPs Circulating ASCs	CD44 + CD56 + [42] CD73 + [147] PDGFRA + CD90 + [148] CD34 + CXCR4 + [91]	Myokines, adipocytokines
**Bone marrow**	MAT	Altered HSCs commitment, altered bone remodeling	MSCs (BM-MSCs)	LepR + [61] Osterix + [62]	Leptin, adiponectin, pro-inflammatory cytokines, osteocalcin, FFAs
**Heart**	EAT, PAT, PVAT	Myocardial dysfunction, HFpEF, CAD	MCs FAPs	PDGFRa + PDGFRb + [90] PDGFRA + [53]	Pro-inflammatory cytokines
**Thymus**	TAT	Reduced T-cell response to antigens	ASCs	CD34 + CD73 + CD90 + [149]	Pro-inflammatory cytokines
**Peritumoral**	CAAs	Tumorigenesis, tumor progression, invasiveness and spreading	ASCs	Not reported	Lipid products, leptin, pro-inflammatory cytokines, VEGF

For each organ (Regional distribution), we reported the acronym of the local AT (Adipose tissue localization (Acronym)), some of the clinical features associated with the increase of AT (Pathological implications), the cells in which adipocytes originate (Adipocyte progenitors) and some of the markers used for their identification (Progenitor markers). In the last column, we reported a list of signalling molecules that are altered when AT increases ectopically

*SAT* subcutaneous AT, *VAT* visceral AT, *BAT* brown AT, *IMAT* intermuscular AT, *IMCL* intramyocellular lipids, *MAT* marrow AT, *EAT* epicardial AT, *PAT* paracardial AT, *PVAT* perivascular AT, *TAT* thymic AT, *CAAs* cancer-associated adipocytes, *IR* insulin resistance *T2D* type 2 diabetes, *NAFLD/NASH* non-alcoholic fatty liver disease/non-alcoholic steatohepatitis, *CVD* cardiovascular diseases, *HSCs* hematopoietic stem cells, *CAD* coronary artery disease, *HFpEF* heart failure with preserved ejection fraction, *ASCs* adipose tissue-derived stromal and stem cells, *MSCs* mesenchymal stromal cells, *SCs* satellite cells, *FAPs* fibro-adipogenic precursors, *BM-MSCs* bone marrow MSCs, *MCs* mesenchymal cells, *FFAs* free fatty acids, *VEGF* vascular-endothelial growth factor
